# Successful Allogeneic Stem Cell Transplant for Philadelphia Chromosome Negative Acute Lymphoblastic Leukemia After Pneumonectomy for Pulmonary Mucormycosis: A Case Report and Review of the Literature

**DOI:** 10.1002/jha2.70125

**Published:** 2025-08-09

**Authors:** Alexander O'Hara, Sanja Zepcan, Stephanie Tsai, Imran Puthawala, Jorgena Kosti‐Schwartz

**Affiliations:** ^1^ Internal Medicine Loyola University Medical Center Chicago Illinois USA; ^2^ Hematology and Bone Marrow Transplantation Loyola University Medical Center Chicago Illinois USA; ^3^ Hematology and Bone Marrow Transplantation Loyola University Chicago Stritch School of Medicine Maywood Illinois USA

## Abstract

The incidence of relapsed Philadelphia chromosome‐negative acute lymphoblastic leukemia in adults is estimated to be 40%–50%. Allogeneic stem cell transplantation can improve survival in relapsed acute lymphoblastic leukemia; however, impaired pulmonary function is detrimental for surviving the transplantation process. Here, we present a successful case of allogeneic transplantation for relapsed B‐acute lymphoblastic leukemia after total resection of the left lung due to angioinvasive pulmonary mucormycosis. To our knowledge, this is the first successful case of allogeneic transplantation after total pneumonectomy. Specific considerations in this case included careful donor selection, judicious choice of pre‐transplantation conditioning regimen, and utilizing novel immunotherapies to ensure major residual disease negativity prior to transplant. It is our hope that this case provides additional guidance to clinicians caring for patients with hematologic malignancies who develop invasive fungal infections and require major lung surgeries.

1

The incidence of relapsed Philadelphia chromosome‐negative acute lymphoblastic leukemia (Ph‐negative B‐ALL) in adults is estimated to be 40%–50%, with contributing factors including disease risk, first‐line chemotherapy regimen, measurable residual disease (MRD) negativity and time to achievement, and consolidation with allogeneic hematopoietic stem cell transplantation (allo‐HCT) [1, 2]. Allo‐HCT confers better long‐term remission rates in relapsed or refractory B‐ALL. In the UKALLXII/ECOG2993 trial, patients with relapsed Ph‐negative B‐ALL who underwent allo‐HCT from a sibling donor had better 5‐year overall survival (23%) compared to patients who received additional chemotherapy alone (4%) [3]. Recently, the addition of blinatumomab, a Bi‐specific T‐cell engager antibody, and inotuzumab ozogomicin, a monoclonal antibody drug conjugate, has been shown to improve MRD negativity rates in relapsed disease [4, 5]. Impaired pulmonary function is detrimental for surviving allo‐HCT. Here, we describe a successful case of allo‐HCT for relapsed Ph‐negative B‐ALL after resection of the left lung due to an invasive fungal infection. Cases of allo‐HCT have been reported after lobectomy for invasive pulmonary fungal infections; to our knowledge, this is the first reported case of a patient who successfully received allo‐HCT after a total pneumonectomy.

**FIGURE 1:**
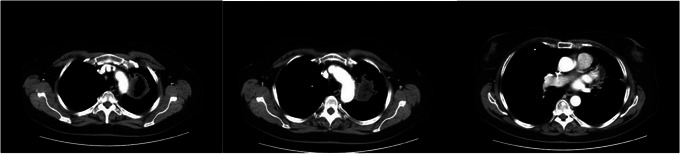
Sections of CT angiography demonstrating left mainstem bronchus involvement, pulmonary artery occlusion, and pericardial involvement of fungal mass.

**FIGURE 2:**
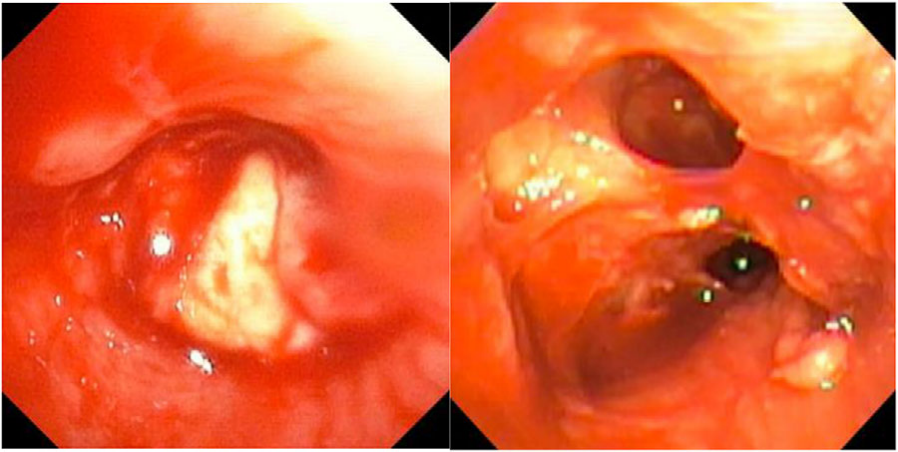
Images from bronchoscopy showing total occlusion of the left mainstem bronchus (left) with partial resolution (right) after removal for pathology analysis by interventional pulmonology.

A 62‐year‐old woman with bipolar I disorder and a prior subarachnoid hemorrhage (SAH) presented to an outside hospital with gingival bleeding. She was found to have B‐ALL. The initial bone marrow biopsy showed hypercellular marrow effaced with leukemic blasts. Karyotype analysis showed t(7:19), t(2:14), and del(9). The fluorescence in situ hybridization (FISH) study was negative for the BCR::ABL translocation. She received four cycles of Hyper‐CVAD, two cycles of part A (cyclophosphamide, vincristine sulfate, doxorubicin hydrochloride, and dexamethasone), and two cycles of part B (methotrexate and cytarabine) and fluconazole as is standard antifungal prophylaxis for all patients. The post‐induction bone marrow biopsy showed morphological remission. MRD by multiparametric flow cytometry with a sensitivity of 10^−5^ was negative and MRD by next‐generation sequencing (NGS) through clonoSEQ was not sent. She was referred to our center for transplant.

During the transplant workup, a CT scan of the chest revealed a perihilar mass obliterating the left pulmonary artery (Figure 1). A trans‐thoracic bronchoscopy revealed a large mass occupying the left mainstem bronchus, and histopathology showed angioinvasive mucormycosis (Figure 2). She was started on isavuconazole, as she carried a prior allergy to posaconazole, and amphotericin B [6]. Disseminated mucormycosis was ruled out. She then underwent successful intrapericardial total extrapleural pneumonectomy, which delayed plans for allo‐HCT. Amphotericin B was stopped and she was maintained on isavuconazole secondary prophylaxis at 372 mg/day.

The patient remained disease‐free without any maintenance or consolidation therapy due to concern for recurrent mucormycosis. Two years later, a repeat surveillance bone marrow biopsy showed 94% blasts consistent with relapsed B‐ALL. The cytogenetics and FISH studies revealed loss of CDKN2A and were negative for the BCR::ABL translocation. Daunorubicin, cyclophosphamide, and pegaspargase were initiated as per CALGB 9511 protocol [7]. Vincristine was held to avoid drug–drug interactions with isavuconazole as recommended by the manufacturer [8]. Intrathecal triple chemotherapy with methotrexate, cytarabine, and hydrocortisone was administered in eight lumbar punctures. Other possible drug interactions with isavuconazole were monitored using a Lexicomp tool integrated into the electronic medical record under the supervision of a transplant pharmacist. A repeat bone marrow biopsy showed no morphological evidence of ALL; however, MRD was positive by NGS through clonoSEQ. She then received two cycles of blinatumomab but MRD by clonoSEQ remained positive. After one cycle of inotuzumab ozogomicin, she achieved negative MRD by flow as well as by colonoSEQ with a sensitivity of 10^−6^.

The patient went on to matched sibling donor allo‐HCT. Antifungal prophylaxis with mold coverage is not routinely administered to patients with Ph‐negative B‐ALL prior to transplant at our institution; this patient continued isavuconazole at 372 mg/day given her prior pneumonectomy. A drug level was found to be therapeutic on admission for transplant but was not routinely monitored. At the time of transplantation, her HCT‐specific comorbidity index (HCT‐CI) score was 5 due to her reduced diffusing capacity of the lungs for carbon monoxide (DLCO) with prior pneumonectomy, as well as her psychiatric history and history of SAH. Her Karnofsky Performance Status (KPS) was 90%.

She received reduced intensity conditioning (RIC) with one dose of thiotepa (5 mg/m^2^), three doses of busulfan (3.2 mg/kg), and four doses of fludarabine (30 mg/m^2^). Isavuconazole was held 5 days prior to receiving busulfan and resumed on Day +1. Her post‐transplant course was complicated by grade 2 acute graft versus host disease (aGVHD) involving the skin and oral mucosa, treated with steroids and ruxolitinib. Day 100 and 6‐month bone marrow biopsies showed remission with negative MRD by flow and clonoSEQ, and post‐transplant chimerisms remained at >98% donor cells.

Mucormycosis is an aggressive disease present mainly in immunocompromised patients with high morbidity and mortality. Several reports describe patients who have undergone successful treatment of hematologic malignancies after lobectomy for invasive fungal infections. One case describes a 59‐year‐old man with acute myelogenous leukemia (AML) who developed a cavitary right lower lobe lesion, treated with amphotericin B, posaconazole, and right lower lobe lobectomy. He underwent successful allo‐HCT after surgery [9]. Another case describes a 59‐year‐old woman with AML who developed a right upper lobe pulmonary mucormycosis infection, treated with amphotericin B and right upper lobe lobectomy. This patient received a successful allo‐HCT from a haploidentical donor [10]. Two larger case series describe cohorts of six patients each who underwent successful allogeneic or autologous stem cell transplant after lobectomy or wedge resection for invasive fungal disease. In all of these cases, the surgical intervention was limited to partial resection of the affected lung, not total pneumonectomy [11, 12]. Our patient also received secondary antifungal prophylaxis with isavuconazole as she had not completed consolidation therapy at the time of her index infection, and the risks of disease relapse and recurrence were high. While there are no consensus guidelines regarding continuing prophylaxis in these situations, there is some evidence that it may be of benefit [13].

Selecting candidates for allo‐HCT requires consideration of a patient's disease risk and control, medical comorbidities, functional status, donor availability, and social support. The HCT‐CI score is a validated tool that risk‐stratifies rates of overall survival and non‐relapse mortality (NRM) based on pre‐transplantation comorbidity burden. Our patient had an HCT‐CI score of 5, corresponding with a predicted 3‐year NRM of 35% and 3‐year overall survival of 38% [14]. She was eligible for allo‐HCT in the setting of an available full‐matched sibling donor and excellent functional status, response to salvage therapy, and social support. The circumstances surrounding this case required careful consideration of the appropriate conditioning regimen and donor selection with priority to preserve remaining pulmonary function and avoid NRM. A fully matched 50‐year‐old sibling donor was selected, a decision centered on performing the transplant in the timeliest manner possible.

In pediatric patients with B‐ALL myeloablative, conditioning regimens with total body irradiation (TBI) are associated with lower rates of relapse (30.6% vs. 49.3%; *p* < 0.00001) and better 5‐year overall survival (58.8% vs. 35.9%; *p* < 0.00001) than regimens without TBI [15]. However, TBI containing myeloablative regimens has been associated with higher rates of chronic graft versus host disease (cGVHD) and its associated pulmonary toxicities compared to regimens containing thiotepa (43% vs. 29%; *p* = 0.03) [16]. In pediatric patients undergoing allo‐HCT, there was no statistically significant difference in rates of relapse (23% vs. 28%; *p* = 0.24), NRM (20% vs. 26%; *p* = 0.61), leukemia‐free survival (57% vs. 46%; *p* = 0.12), and overall survival (67% vs. 56%; *p* = 0.18) when TBI‐based regimens were compared to thiotepa‐based regimens prior to allo‐HCT [15]. These data are frequently extrapolated to the adult population and prompted our selection of a thiotepa‐based regimen. Our patient received RIC with thiotepa, busulfan, and fludarabine to reduce the risk of busulfan‐induced pulmonary toxicity [17]. Isavuconazole was held 5 days prior to busulfan to reduce the risk of metabolic interactions including hepatic veno‐occlusive disease. The toxicities associated with myeloablative TBI‐based regimens have been considered a barrier to offering allo‐HCT to older and frail patients with B‐ALL. Thiotepa‐based RIC regimens could increase access to transplant for these patients.

At the same time, to maximize odds of long‐term survival in this patient, we sought total MRD negativity prior to transplant. Blinatumomab has been shown to induce MRD negativity in 76%–82% of patients with relapsed or refractory Ph negative B‐ALL and is now often used as a bridge to transplant [4, 18]. Our patient remained MRD positive by NGS after two cycles of blinatumomab for which she then received one cycle of the monoclonal antibody‐cytotoxic drug conjugate inotuzumab ozogamicin. This agent has also been associated with high MRD negativity rates in MRD‐positive B‐ALL patients. In a recent phase II trial, 18 out of 26 MRD‐positive patients (69%) achieved MRD negativity after receiving between one and six (median 3) cycles of inotuzumab [5]. After two cycles of blinatumomab and one cycle of inotuzumab, our patient achieved MRD negativity prior to transplant. Our patient remains in remission 1‐year post‐transplant at the time of this writing. She has continued isavuconzole antifungal prophylaxis without signs of cardiopulmonary complications. She has grade II aGVHD of the skin and moderate total cGVHD with favorable response to ruxolitinb.

Transplant can be curative in relapsed Ph‐negative B‐ALL but is associated with significant morbidity and mortality in individuals with comorbidities and diminished baseline organ function. Despite this patient's prior pneumectomy, she was able to successfully receive allo‐HCT with careful donor selection, judicious choice of conditioning, and utilizing novel immunotherapies to ensure MRD negativity. Our approach and conclusions are limited by their application to a single case as of this writing. It is our hope that this case provides additional guidance to clinicians caring for patients with hematologic malignancies who develop invasive fungal infection and require major lung surgeries.

## Author Contributions

Alexander O'Hara, Sanja Zepcan, Stephanie Tsai, Imran Puthawala, and Jorgena Kosti‐Schwartz. wrote and edited the paper.

## Ethics Statement

The authors have nothing to report.

## Consent

The authors have nothing to report.

## Conflicts of Interest

S.J. is a speaker for Sanofi. S.T. has received honoraria from Sanofi, Jazz Pharmaceuticals, and Bristol Meyers Squibb, as well as consulting fees from Gamida Cell. All other authors declare no conflicts of interest.

## Data Availability

Data sharing is not applicable to this article as no new data were created or analyzed in this study.

## References

[1] M. S. Schwartz and L. S. Muffly , “Predicting Relapse in Acute Lymphoblastic Leukemia,” Leukemia & Lymphoma 65, no. 13 (2024): 1934–1940. 39216505 10.1080/10428194.2024.2387728

[2] K. Sasaki , E. Jabbour , N. J. Short et al., “Acute Lymphoblastic Leukemia: A Population‐Based Study of Outcome in the United States Based on the Surveillance, Epidemiology, and End Results (SEER) Database, 1980–2017,” American Journal of Hematology 96, no. 6 (2021): 650–658. 33709456 10.1002/ajh.26156 PMC9517941

[3] A. K. Fielding , S. M. Richards , R. Chopra , et al., “Outcome of 609 Adults after Relapse of Acute Lymphoblastic Leukemia (ALL); an MRC UKALL12/ECOG 2993 Study,” Blood 109, no. 3 (2007): 944–950. 17032921 10.1182/blood-2006-05-018192

[4] F. Haddad , H. Kantarjian , N. J. Short , et al., “A Phase II Study of Blinatumomab for the Treatment of Measurable Residual Disease‐Positive B‐Cell Acute Lymphoblastic Leukemia,” Blood 138 (2021): 4398.

[5] E. Jabbour , F. G. Haddad , N. J. Short , et al., “Phase 2 Study of Inotuzumab Ozogamicin for Measurable Residual Disease in Acute Lymphoblastic Leukemia in Remission,” Blood 143, no. 5 (2024): 417–421. 37879077 10.1182/blood.2023022330

[6] Referenced with Permission from the NCCN Clinical Practice Guidelines in Oncology (NCCN Guidelines®) for Prevention and Treatment of Cancer Related Infections V32024 © National Comprehensive Cancer Network, Inc 2024 All Rights Reserved. Accessed 5/8/2025. To view the most recent and complete version of the guideline, go online to NCCN.org.

[7] M. Wetzler , B. L. Sanford , J. Kurtzberg , et al., “Effective Asparagine Depletion With Pegylated Asparaginase Results in Improved Outcomes in Adult Acute Lymphoblastic Leukemia: Cancer and Leukemia Group B Study 9511,” Blood 109, no. 10 (2007): 4164–4167. 17264295 10.1182/blood-2006-09-045351 PMC1885493

[8] CRESEMBA [package insert]. Northbrook, IL: Astellas Pharma US, Inc.

[9] A. Amin and M Y. Chow , “Pulmonary Mucormycosis Treated with Combination Antifungals and Lobectomy Prior to Stem Cell Transplant in a Patient with AML,” Chest 156, no. 4 (2019): A629.

[10] K. Miura , N. Kobayashi , I. Ito , et al., “Pulmonary Mucormycosis Developed during Acute Myelogenous Leukemia and Successfully Treated by Surgical Resection before Blood Stem Cell Transplantation,” AME Case Reports 3, no. 48 (2019): 1–4. 32030366 10.21037/acr.2019.11.02 PMC6987314

[11] N. Harada , S. I. Kimura , A. Gomyo , et al., “Surgical Resection for Persistent Localized Pulmonary Fungal Infection Prior to Allogeneic Hematopoietic Stem Cell Transplantation: Analysis of Six Cases,” Journal of Infection and Chemotherapy 26, no. 2 (2020): 175–180. 31735628 10.1016/j.jiac.2019.08.003

[12] A. Nosari , M. Ravini , R. Cairoli , et al., “Surgical Resection of Persistent Pulmonary Fungus Nodules and Secondary Prophylaxis Are Effective in Preventing Fungal Relapse in Patients Receiving Chemotherapy or Bone Marrow Transplantation for Leukemia,” Bone Marrow Transplantation 39, no. 10 (2007): 631–635. 17384656 10.1038/sj.bmt.1705655

[13] F. Danion , A. Coste , C. Le Hyaric , et al., “What Is New in Pulmonary Mucormycosis?,” Journal of Fungi 9, no. 3 (2023): 307. 36983475 10.3390/jof9030307 PMC10057210

[14] M. L. Sorror , M. B. Maris , R. Storb , et al., “Hematopoietic Cell Transplantation (HCT)‐Specific Comorbidity Index: A New Tool for Risk Assessment Before Allogeneic HCT,” Blood 106, no. 8 (2005): 2912–2919. 15994282 10.1182/blood-2005-05-2004 PMC1895304

[15] F. Ansari , M. Behfar , L. Jafari , et al., “A Comprehensive Comparison Between TBI vs Non‐TBI‐Based Conditioning Regimen in Pediatric Patients with Acute Lymphoblastic Leukemia: A Systematic Review and Meta‐Analysis,” Leukemia Research 135 (2023): 107416. 37918224 10.1016/j.leukres.2023.107416

[16] G. Battipaglia , M. Labopin , S. Mielke , et al., “Thiotepa‐based Regimens Are Valid Alternatives to Total Body Irradiation‐Based Reduced‐Intensity Conditioning Regimens in Patients With Acute Lymphoblastic Leukemia: A Retrospective Study on Behalf of the Acute Leukemia Working Party of the European Society for Blood and Marrow Transplantation,” Transplantation and Cellular Therapy 30, no. 1 (2024): 95. e1. 10.1016/j.jtct.2023.09.028 37816471

[17] A. De Masson , M. Beylot‐Barry , C. Ram‐Wolff , et al., “Allogeneic Transplantation in Advanced Cutaneous T‐Cell Lymphomas (CUTALLO): A Propensity Score Matched Controlled Prospective Study,” Lancet 401, no. 10392 (2023): 1941–1950. 37105210 10.1016/S0140-6736(23)00329-X

[18] E. Curran and W. Stock , “Taking a “BiTE out of ALL”: Blinatumomab Approval for MRD‐positive ALL,” Blood 133, no. 16 (2019): 1715–1759. 30796026 10.1182/blood-2018-12-852376 PMC6634959

